# Retrospective analysis of remifentanil combined with dexmedetomidine intravenous anesthesia combined with brachial plexus block on shoulder arthroscopic surgery in elderly patients

**DOI:** 10.12669/pjms.38.6.5724

**Published:** 2022

**Authors:** Yan Zhang, Lingling Zhao, Liangce Lv, Songxue Li

**Affiliations:** 1 Yan Zhang, Department of Anesthesiology, Jiading Branch of Shanghai General Hospital, Shanghai Jiao Tong University School of Medicine, 800 Huangjiahuayuan Road,Shanghai 201803, P.R. China; 2 Lingling Zhao, Department of Anesthesiology, Jiading Branch of Shanghai General Hospital, Shanghai Jiao Tong University School of Medicine, 800 Huangjiahuayuan Road,Shanghai 201803, P.R. China; 3 Liangce Lv, Department of Anesthesiology, Jiading Branch of Shanghai General Hospital, Shanghai Jiao Tong University School of Medicine, 800 Huangjiahuayuan Road,Shanghai 201803, P.R. China; 4 Songxue Li, Department of Anesthesiology, Jiading Branch of Shanghai General Hospital, Shanghai Jiao Tong University School of Medicine, 800 Huangjiahuayuan Road,Shanghai 201803, P.R. China

**Keywords:** Remifentanil, Dexmedetomidine, Combined intravenous anesthesia, Brachial plexus block, Arthroscopic shoulder surgery

## Abstract

**Objectives:**

To analyze the effect of remifentanil combined with dexmedetomidine intravenous anesthesia combined with brachial plexus block on shoulder arthroscopic surgery in elderly patients.

**Methods:**

This retrospective study conducted at Jiading Branch of Shanghai General Hospital, investigated clinical data from elderly patients receiving shoulder arthroscopy between January 2020 and June 2021. Based on the treatment, patients were retrospectively divided into Group-I (remifentanil combined with dexmedetomidine) and Group-II (remifentanil continuous pump injection). Hemodynamic indices, such as mean arterial pressure (MAP) and heart rate (HR), degree of pain (VAS score), and stress response marker levels were examined prior to the operation and at various time points post-operation. Operation time and adverse reaction incidences were also evaluated.

**Results:**

There was no significant differences in MAP and HR between the two groups prior to the operation. However, MAP and HR levels were lower in Group-I patients at three time points post-operation. Similarly, VAS scores were not different between the two groups prior to the operation but were much lower in Group-I at multiple time points post-operation. The same trend was observed for the stress-induced angiotensin-II, cortisol, and aldosterone. Additionally, patients in Group-I had lower incidence of adverse reactions and shorter operation time.

**Conclusion:**

Remifentanil combined with dexmedetomidine intravenous anesthesia for shoulder arthroscopic surgery under general anesthesia combined with brachial plexus block in elderly patients can maintain hemodynamic stability, shorten operation time, reduce the degree of stress reaction, pain caused by invasive operation, and reduce the incidence of adverse reactions.

## INTRODUCTION

Arthroscopy is commonly used in the clinical treatment of shoulder diseases and injuries, and is associated with lower incidence of trauma, fewer complications, and faster postoperative functional recovery.^1^ However, a specific posture such as lateral traction posture, as well as continuous pressure cleaning of the joint cavity are usually required during shoulder arthroscopic surgery. Furthermore, a controlled hypotension intervention is administered to reduce blood loss and ensure a clear operative field. The large amounts of flushing during the operation may cause upper respiratory tract obstruction and tracheal compression. As a result, general anesthesia is usually employed.^2^,^3^ Brachial plexus block is often used during shoulder arthroscopic surgery due to its relatively small impact on body function. However, there is always a risk of incomplete block.^4^,^5^ It is therefore important to investigate how to improve sedation and analgesia for patients undergoing shoulder arthroscopy.

Remifentanil, an ultra-short opioid analgesic, offers antihypertensive as well as analgesic effects. However, elevated dosages of this agent may increase the risk of respiratory depression.^6^ By contrast, dexmedetomidine hydrochloride is a new analgesic sedative that elicits no respiratory inhibition while inhibiting sympathetic activity. As such, it can effectively regulate heart rate and blood pressure, and therefore plays an important role in the treatment of many diseases.^7^,^8^ The main goal of our study was to investigate the efficacy of remifentanil combined with dexmedetomidine intravenous compound anesthesia in elderly patients undergoing shoulder arthroscopy.

## METHODS

Clinical records of elderly patients who received shoulder arthroscopy in our hospital from January 2020 to June 2021 were retrospectively selected. There was a total of 96 patients (54 males and 42 females). According to different anesthesia methods, they were retrospectively divided into two groups. Patients that received remifentanil combined with dexmedetomidine intravenous anesthesia combined with brachial plexus block were set as Group-I (n=48) and patients that received remifentanil intravenous anesthesia combined with brachial plexus block were set as Group-II (n=48).The ethic committee of Jiading Branch of Shanghai General Hospital (Approval number: 2021027, Date: March 27, 2021).

### Inclusion Criteria

Patients fit the criteria qualifying them for shoulder arthroscopy (For example: rotator cuff injury; Instability of shoulder joint; Tear of glenoid lip of shoulder joint; subacromial impingement syndrome); age of 60 years or older; American Society of Anesthesiology(ASA) grade of received anesthesia of II-III; complete clinical data available.

### Exclusion Criteria

Patients with atrioventricular block and bradycardia; patients with benign and malignant tumors; psychosis; abnormal organ function; cardiovascular and cerebrovascular diseases, including abnormal coagulation function or circulatory system lesions; infectious diseases and puncture site infections.

The treatment protocol was as follows. First, the peripheral venous channels were opened in the healthy upper limbs, and bispectral index (BIS) value, arterial oxygen saturation (SpO2), blood pressure, and ECG monitoring commenced. Patients in Group-II were continuously administered remifentanil at a dose of 0.05ug/(kg·min). Patients in Group-I were continuously administered remifentanil 0.05ug/(kg·min) combined with dexmedetomidine 0.5ug/kg. Anesthesia induction scheme was identical for both groups: intravenous injection of sufentanil(0.5ug/kg), propofol(0.3mg/kg), and cisatracurium(0.2mg/kg). Patients underwent endotracheal intubation and were ventilated at a rate of 10-12times/minute with a tidal volume of 6-8ml/kg. Ten minutes after intravenous administration, brachial plexus block was performed to assist the patient in taking the prone position. The skin was disinfected, and a sterile fiber tube sleeve was placed on a linear array ultrasonic probe (8-14mHz) and was positioned on the clavicle of the affected side. The brachial plexus was clarified between the scalene muscles, and the nerve stimulation needle was connected. If shoulder and upper arm convulsions could be induced at 0.3mA, 15ml ropivacaine (0.25%) was injected between the anterior and middle scalene muscles (which could be visualized ultrasonically as an elliptic diffusion. Maintenance anesthesia was comprised of sufentanil (3-5ug) and cisatracurium (3mg) administered intermittently based on the operation duration. All patients were treated with patient-controlled intravenous analgesia (PCIA). The drug formula was as follows: sufentanil 2ug/kg, tropisetron 10mg, sodium chloride injection diluted to 100ml. Parameter settings were: background dose 2ml/hour, single press dose 2ml, locking time 15minutes.

Vital sign monitoring was used to record hemodynamic index levels-including mean arterial pressure (MAP) and heart rate (HR) at three time points: prior to the operation(T0), during the cutting of the skin(T1), 30 minutes after the start of the operation(T2), and at the end of the operation(T3). Pain was evaluated prior to the operation and at the following time points after the operation: immediately, two hours, six hours and twelve hours. Pain was assessed using the VAS scale(from 0-10, with higher scores indicating more serious pain levels). Stress response indices at T0, T3, 12 hours post operation(T4), and 24 hours post-operation(T5), including angiotensin II(Ang-II), cortisol(COR), and aldosterone(ALD) levels, were measured using radioimmunoassay (The kit is provided by Beijing atomic high tech nuclear technology application Co., Ltd. and the detection instrument is GC-400 γ-radioimmunoassay counter.). Adverse reaction incidence (such as Shivering, Bradycardia, Nausea and Vomiting, Cough, respiratory depression, etc.) and operation time were recorded. Data were analyzed using SPSS v.22.0. Measurement data were expressed as (*X̅*±S) and compared using t-tests. The count data was expressed as n(%) and examined via *Χ^2^* tests, *P*<0.05 indicates a statistically significant difference.

## RESULTS

The study included a total of 96 patients, 54 males and 42 females. Group-I patients (48) contained 28 males and 20 females, with the age range of 63~86 years (average: 72.22±5.32 years). According to the physical condition classification of American Society of Anesthesiologists (ASA),^9^ 28 patients were classified as class II and 20 patients were classified as class III. The average body mass index (BMI) of the patients in Group-I ranged from 17.5 to 26.8 kg/m^2^ (average: 22.78±2.71 kg/m^2^). This group comprised 25 cases of rotator cuff injury, nine cases of shoulder instability, six cases of glenoid lip tear, and eight cases of subacromial impingement syndrome.

Group-II included 48 patients, 26 males and 22 females, with the age ranging from 62 to 84 (71.43±5.91) years. There were 27 patients with ASA class II classification and 21 patients with ASA class III classification. BMI ranged between 17.4-28.1 kg/m^2^ (average: 22.35±2.73 kg/m^2^). This group included 28 cases of rotator cuff injury, eight cases of shoulder instability, three cases of glenoid lip tear, and nine cases of subacromial impingement syndrome. No differences were noted between groups in terms of gender, age, ASA grade, BMI, and disease composition.

There were no differences in MAP and HR levels between the two groups at T0. However, Group-I MAP and HR values were lower than those of Group-II at T1, T2, and T3 (P<0.05, Table-I). Similarly, no differences in VAS score were noted between the two groups prior to the operation. However, VAS scores in Group-I were significantly lower than in Group-II immediately after the operation, as well as two hour, six hour, and 12 hour after the operation (P<0.05, Table-II).

**Table I:** Comparison of hemodynamic indices (*X̅*±S).

Index	Group	n	T_0_	T_1_	T_2_	T_3_
MAP (mmHg)	Group-I	48	77.43±10.35	85.10±11.66	85.16±11.92	84.18±11.32
	Group-II	48	80.02±10.19	91.56±12.36	91.16±12.12	89.16±9.81
	t		1.231	2.632	2.445	2.302
	P		0.221	0.010	0.016	0.024
HR (time/min)	Group-I	48	75.85±8.91	78.87±9.50	82.72±10.10	78.43±9.62
	Group-II	48	77.33±9.32	84.79±11.30	87.89±11.68	84.18±10.82
	t		0.795	2.775	2.318	2.751
	P		0.429	0.007	0.023	0.007

**Table II:** Comparison of VAS scores (*X̅*±S).

Group	n	Preoperative	Immediately after operation	2hours after operation	6h after operation	12h after operation
Group-I	48	6.29±1.25	2.52±0.77	2.75±0.97	3.04±1.03	2.50±0.79
Group-II	48	6.70±1.24	3.47±1.05	3.64±1.08	4.29±1.21	3.08±1.08
t		1.628	5.090	4.255	5.424	2.994
P		0.107	<0.001	<0.001	<0.001	0.004

In terms of stress response, there was no significant difference in serum Ang-II, COR, and ALD levels between the two schemes at T0. However, serum Ang-II, COR, and ALD levels in Group-I patients were significantly lower than those of Group-II patients at T3, T4, and T5 (P<0.05, Table-III). There were fewer adverse reactions in Group-I patients (6.25%) compared to Group-II patients (20.83%) (P<0.05, Table-IV), and the operation time in Group-I patients (59.91±5.35 minutes) was shorter as compared to Group-II patients (65.28±7.11 minutes) (t=4.181, P<0.001).

**Table III:** Comparison of stress response index levels (*X̅*±S), μg/L).

Index	Group	n	T_0_	T_3_	T_4_	T_5_
Ang-II	Group-I	48	39.27±8.84	44.04±9.40	46.93±9.88	41.60±9.48
	Group-II	48	38.81±8.06	52.91±9.31	63.12±9.63	57.45±9.60
	t		0.265	4.646	8.125	8.136
	P		0.791	<0.001	<0.001	<0.001
Cor	Group-I	48	22872±33.67	242.89±34.89	257.91±38.06	266.43±34.99
	Group-II	48	223.14±27.25	267.37±32.71	313.39±37.34	296.60±31.99
	t		0.893	3.546	7.208	4.408
	P		0.374	0.001	<0.001	<0.001
ALD	Group-I	48	200.33±23.72	215.04±26.63	231.31±28.42	221.12±26.27
	Group-II	48	204.12±20.32	233.04±22.57	265.45±25.89	272.87±28.61
	t		0.841	3.572	6.153	9.229
	P		0.403	0.001	<0.001	<0.001

**Table IV:** Comparison of adverse reaction rates [n (%)].

Group	n	Shivering	Bradycardia	Nausea and Vomiting	Cough	Respiratory depression	Total incidence
Group-I	48	1(2.08)	0(0.00)	1(2.08)	1(2.08)	0(0.00)	3(6.25)
Group-II	48	1(2.08)	1(2.08)	3(6.25)	3(6.25)	2(4.17)	10(20.83)
Χ^2^							4.360
P							0.037

## DISCUSSION

This study retrospectively analyzed the use of remifentanil and dexmedetomidine in shoulder arthroscopic surgery under general anesthesia combined with brachial plexus block in elderly patients. The study found that MAP and HR levels, VAS scores, and stress indices were all lower in Group-I patients compared to Group-II. This shows that the use of remifentanil and dexmedetomidine can inhibit sharp hemodynamic fluctuations, shorten operation times, reduce stress, and effectively alleviate postoperative pain. Therefore, this strategy has high applicative value in shoulder arthroscopic surgery.

Dexmedetomidine and remifentanil are both analgesic sedatives. It has been shown that low dexmedetomidine concentrations can induce sedation, and that sensitivity to this agent increases with age.^10^ Research has also shown that dexmedetomidine combined with remifentanil is safe and efficacious for lower eyelid plasty,^11^ and is associated with significantly reduced intubation times, improved arterial pressure during extubation, lower heart rate and VAS scores, and improved quality of postoperative analgesia. Patient and surgeon satisfaction was recorded as 96%. The results of our study are, therefore, consistent with these observations.

Dexmedetomidine activates spinal cord α2 adrenoceptors, blocking neuronal discharge and resulting in analgesia.^12^,^13^ These α2 adrenoceptor-mediated negative feedback mechanisms can regulate ATP and norepinephrine production, reduce neuronal excitability, and strengthen the analgesic function of remifentanil.^14^,^15^ Moreover, dexmedetomidine can inhibit the sympathetic reflex caused by invasive operations, strengthen vagus nerve activity, and affect the presynaptic function of sympathetic nerve terminals that can be stimulated by α2 receptors. This leads to downregulation of plasma catecholamines, reduces stress responses and stabilizes hemodynamics.^16^,^17^ Dexmedetomidine binding to α2 receptors can also reduce adenylate cyclase activity, reduce cyclic adenosine monophosphate synthesis, inhibit calcium channels and calcium ionic influx to nerve terminals, prevent transmitter generation, cause presynaptic membrane hyperpolarization, thereby inducing sedation.

This study also found that the incidence of adverse reactions in Group-I was lower than that in Group-II. Our results are in agreement with previous studies. Abdalla W et al.^18^ found that the combination of dexmedetomidine and remifentanil offered a synergistic reduction of analgesic demand and resulted in fewer maternal and neonatal adverse events compared to remifentanil alone. The administration of dexmedetomidine can also reduce the necessary dosage of other general anesthesia agents, further reducing rate of potential adverse reactions. In a randomized controlled trial that included 189 patients, Lu Z et al.^19^ observed that the respiratory effect and satisfaction score of patients that received dexmedetomidine combined with remifentanil were significantly higher than those in the midazolam remifentanil group. Furthermore, operation time in patients that were administered dexmedetomidine combined with remifentanil was shorter, similar to the findings of this study.

### Limitations

This is a retrospective, single-center study with a small number of patients. Further prospective, multi-center studies in larger cohorts are needed. Additionally, it is not clear presently if this combined anesthesia scheme will have a positive impact on the functional rehabilitation of patients undergoing shoulder arthroscopic surgery. More studies are needed to evaluate this outcome.

## CONCLUSION

Remifentanil combined with dexmedetomidine intravenous anesthesia in shoulder arthroscopic surgery under general anesthesia combined with brachial plexus block in elderly patients can maintain hemodynamic stability, shorten operation duration, reduce stress reactions and pain, and yield lower incidence of adverse reactions.

### Authors’ contributions:

**YZ:** Conceived and designed the study.

**LZ, LL & SL:** Collected the data and performed the analysis.

**YZ:** Was involved in the writing of the manuscript and is responsible for the integrity of the study.

All authors have read and approved the final manuscript.
